# Old Eyes Made New

**Published:** 1874-02

**Authors:** Charles A. Robertson


BUFFALO

### Medical and Surgical Journal.

VOL XIII. FEBRUARY, 1874. No. 7.

### Original Communications.

:o:

ART. I.—*Old Eyes made New.* By CHARLES A. ROBERTSON, M. D. A paper read before the Albany County Medical Society, January 14th, 1874.

MR. PRESIDENT:—I present to your observation a picture cut from an advertisement, representing a cup about to be applied over the eye of a person by the hand which holds an elastic india-rubber ball. Below you may read the glowing exclamations, printed in capitals with exclamation points to match.

"RESTORE YOUR SIGHT! SPECTACLES AND SURGICAL OPERATIONS RENDERED USELESS! THE INESTIMABLE BLESSING OF SIGHT IS RENDERED PERPETUAL BY THE USE OF THE NEW PATENT IMPROVED EYE-CUP." ABOVE IN DISPLAYED TYPE, IT IS ANNOUNCED THAT MRS. REV. HENRY WARD BEECHER AFTER USING THE IVORY EYE-CUPS, ORDERS A PAIR FOR THE WIFE OF REV. CHARLES BEECHER, OF GEORGETOWN, MASS."

I deem it a duty to utter a warning against the employment of this cupping apparatus as not only a sheer imposition, since it is false in principle, but as also exceedingly dangerous to the well-being of the eye.

It is an old contrivance for the purpose of gain, and palmed off regardless of the consequences that dupes may suffer. A few years ago, an advertisement appeared in the Journals to this effect: "Old eyes made new"—Send for a pamphlet. Price ten cents."

The ten cents would bring back a pamphlet, containing a description of the cups, stating how the eye, flattened by age, could be restored to its pristine state, and giving numerous testimonials of their efficacy. Of course, these were all as false as gravestones. I was told that these cups were made at a factory, at or near Hudson. The pair which I saw were turned out of wood. Instead of the single cup and clumsy hollow ball to exhaust the air, represented in our picture, which can cup only one eye at a time, there were two little wooden cups, each furnished with a small piece of india-rubber tubing that connected in a Y shape with a larger tube. The rejuvenator of his eyes was required to place the main tube in his mouth, apply the cups over his senile eyes and then suck away. It is hardly necessary in this Society to dwell on the dangerous congestion of the delicate structures of the eye, inevitably incident to this dry-cupping operation, to the liability of rupturing delicate blood vessels within the eye-ball, or to mention that, (all risks being set aside from consideration) the convexity of the cornea, which could be produced, must inevitably be very transient and the reaction to its former shape very rapid. You remember the old Roman proverb—"drive out nature with a fork and she will come running back!"

The idea, that the eye-ball possessed such plasticity that it could be altered in form by external force being applied, was conceived by Benjamin Franklin. He advised pressure with the thumb and fingers around the eye-ball so as to force the flattened front of the eye, or cornea, into a greater degree of convexity and by consequence increase its refractive power and obviate the necessity of spectacles. There were, however, two serious errors in the premises from which Franklin reasoned. One error was the untenable assumption that the cornea can be moulded into convexity, so as to practically compensate presbyopia or old sight. The other was the notion that the sight fails in advancing years from a flattening of the eye.

This latter notion still remains in the popular mind, and it is also generally supposed that near-sightedness is the opposite of old-sight (some times called far-sight,) and being due to excess of convexity that the defect of near sight will diminish as age advances. Although occupying still a place in text-books of natural philosophy these notions are to-day demonstrably erroneous.

It has been proved by actual measurement that the cause of near-sightedness is the too great length of the eye from front to back, and a consequent formation of the visual image in front of the retina instead of upon it. In order to throw the image further back a concave glass to diverge the rays of light is required. This defect is therefore structural or anatomical.

Not so, however, in presbyopia. The sight of those who require spectacles for reading or sewing, does not fail for perceiving distant objects. It is only in looking at small objects near by that the necessity for glasses is felt. If the small object is near by, the eye refuses to focus or accommodate so as to make an image on the retina, as in youth. If it is removed to a great distance a very small object will not form in the eye a sufficiently large image to excite visual sensibility. The long mooted question as to where the power of focusing or accommodating for different distances was situated, has been incontrovertibly settled, and it has been conclusively shown, that varying degrees of convexity of the crystalline lens and almost entirely of its anterior face, determine the accommodation. Helmholtz, by a delicate instrument measured the image of a candlelight reflected on the front surface of the lens, and found it greater when the eye was regarding distant objects than when regarding near ones; as the size of an image made by a convex mirror is in inverse ratio to the degree of curvature, it follows from the experiment of Helmholtz, that the anterior reflecting surface of the crystalline lens is less convex in regarding distant objects than when accommodated for near objects. Helmholtz also found that no change took place in the size of the corneal image whether reflected from the cornea while vision was accommodated for a near or a distant point. When the lens is wanting, no accommodation exists. It has been determined by careful observation of many thousand cases, that the power of accommodating for a near point sensibly diminishes from the age of ten years, as the lens becomes firmer, until about the age of forty, it cannot achieve sufficient convexity for accurate definition of fine objects close at hand, and the necessity arises for augmenting the refraction artificially, in other words, for using convex glasses, the ordinary spectacles of elderly persons.

From these considerations it is clear how radically erroneous is the theory that presbyopia may be cured by modifying the convexity of the eye or cornea rather; and it is also clear, that failing sight is owing to a physiological change or hardening of the lens which begins at an early age.

I have said that these eye-cups are dangerous, and that warning should be given against their employment. In proof of this, I will narrate a single case.

Mrs. B. the wife of a wealthy gentleman in a city not very remote, found her sight failing. She did not like to resort to the use of glasses, and having seen the eye-cups advertised as a sure cure she tried them. After a short time the sight of one eye was lost. Thereupon she consulted an oculist of high repute in another city, who examined her eye with lenses and artificial light, and stated to her that she had a cataract. Although she was enjoined to bear her affliction with resignation, she settled down into a state of melancholy on her return home. I chanced to spend a Sunday with friends in the city where she resided, and her husband requested me to ride out to his residence, and if possible cheer her up. Assuming the diagnosis given as a foregone conclusion I was a good deal perplexed as to what I could say to cheer her up; but after having engaged in conversation with her, I requested her to give me the history of her case. She stated that her sight was good until within a few weeks, except that she felt the need of glasses, and she went on to speak of using the eye-cups. One day, she said a black spot appeared before her right eye, and almost immediately it seemed to shoot out processes like a spiders' legs, and her sight was soon gone. This history the gentleman alluded to, had not sought, or I am sure he would have hesitated before comnitting himself to the diagnosis of cataract.

I was glad to find in her account of the manner of losing her vision some ground for encouraging and cheering her. Without even troubling her to submit to examination of the eye, which she seemed to dread lest it should only confirm the opinion already received, I assured her that I was confident that my friend had made a mistake, and that she had no cataract, for her history of the case and the manner of invasion of her blindness negatived the probability of it. I gave my opinion that by use of the dangerous eye-cups she had ruptured a small blood vessel and that the appearance of the spider-like shot was due to intraocular hemorrhage, which I believed would be absorbed and her sight return. I advised her to keep quiet in a shaded apartment, give her eyes absolute functional rest, and to throw her eye-cups into the fire. I had the gratification to learn by letter from her husband within three or four weeks after that her sight had completely returned.

I regarded this as a fortunate escape for her, for it is not always that the effects of intraocular hemorrhages disappear so completely.

